# Modified overlapping suture technique for the repair of horse-tail-like achilles tendon tears: technical description and clinical results

**DOI:** 10.3389/fsurg.2026.1841574

**Published:** 2026-06-15

**Authors:** PengKun Yu, Yu Wu, Qiang Xu, XiaoHong Fan

**Affiliations:** 1 Wenjiang Traditional Chinese Medicine Hospital of Chengdu, Chengdu, China; 2 Chengdu University of Traditional Chinese Medicine, Chengdu, China

**Keywords:** achilles tendon rupture, bunnell suture, horse-tail, krachow, technical description

## Abstract

**Objective:**

To discuss the clinical efficacy of the modified overlapping suture technique to treat horse-tail-like Achilles tendon tears.

**Methods:**

A retrospective analysis was conducted on data from 57 patients with horse-tail-like Achilles tendon tears treated with the overlapping suture technique from May 2020 to May 2024. Calculate the final Arner-Lindholm scores and compare the AOFAS scores, maximum plantar flexion angle, and dorsiflexion angle of the ankle joint before and after surgery, and record the muscle strength of the plantar flexor muscles of the ankle joint and compare it with that before the surgery.

**Results:**

On average, 57 patients completed follow-up within 2 years. One patient experienced delayed wound healing after surgery, two patients re-ruptured their Achilles tendons three weeks post-surgery due to accidental falls, and one patient had a contralateral Achilles tendon rupture 2–4 years post-surgery. In addition, there were no symptoms of peroneal nerve injury after the surgery. The excellent and good rate of the Arner-Lindholm score after surgery was 98.2%; the postoperative AOFAS scores were (92.56 ± 6.71) points, (95% CI, 90.81–94.30), which showed a significant difference compared to the preoperative score of (64.92 ± 9.08) points, (95% CI, 62.56–67.27) (*p* 0.00<0.01). The muscle strength of the plantar flexor group in the affected ankle joint post-surgery was (4.62 ± 1.7) grades (95% CI, 4.17–5.06), which showed no statistical significance compared to the healthy side (4.80 ± 1.3) grades (95% CI, 4.46–5.13) (*p* 0.527 > 0.05); however, there was a significant difference compared to the pre-surgery muscle strength of the plantar flexor group on the affected side (3.2 ± 0.7) grades (95% CI, 3.01–3.8) (*p* 0.00 < 0.01). In the final follow-up, the maximum plantar flexion angle of the affected ankle joint post-surgery was (37.1 ± 3.1)° (95% CI, 36.2–37.9)°, which showed no statistical significance compared to the maximum plantar flexion angle of the healthy side (38.3 ± 3.9)° (95% CI, 37.2–39.2)° (*p* 0.072 > 0.05); the dorsiflexion angle of the ankle joint post-surgery was (18.3 ± 2.2)° (95% CI, 17.7–18.8)°, which showed no statistical significance compared to the dorsiflexion angle of the healthy side (18.2 ± 1.4)° (95% CI, 17.8–18.5)° (*p* 0.773 > 0.05).

**Conclusions:**

The modified overlapping suture technique can be used to treat patients with horse-tail-like Achilles tendon tears. After treatment, the motor function of their lower limbs can be restored. In addition, there are a few complications.

## 1 Introduction

Achilles tendon rupture is a common clinical injury. Spontaneous closed Achilles tendon rupture usually happens 2–6 cm above the Achilles tendon insertion, mainly shown as horse-tail-like tears (1). After a horse-tail-like Achilles tendon tear, the broken ends retract, and thus congestion forms between them, leading to a lack of effective contact. Thus, the effect of the non-surgical treatment is poor (2). After horsetail-like tears, the tendon bundles of the broken end interlace, and the tendon bundles vary in thickness and length; thus, it is prone to slippage and tearing if there is not enough tissue to hold the suture. Subsequently, the Achilles tendon may lengthen or re-rupture, leading to the failure of the surgery (3). If the horse-tail-like Achilles tendon is removed or the broken ends are anastomosed directly, it will result in an Achilles tendon defect and shortening. How to restore the normal length, elasticity, and strength of the Achilles tendon and how to avoid stretching and lengthening of the Achilles tendon after surgery are still challenges for this type of surgery. Younger patients are prone to surgical treatment to achieve firm healing and good functional recovery.

There are two types of surgeries: extensive incision surgery and minimally invasive percutaneous surgery. Although the former can ensure full Achilles tendon alignment, this can easily lead to postoperative infection and delayed loading due to excessive damage to the peritendon blood supply during the operation, ultimately resulting in delayed healing of the injury. Minimally invasive percutaneous surgery can effectively preserve the blood supply to the peritendon tissues, with a lower incidence of postoperative infection and delayed healing. However, during the surgery, it's easy to damage the sural nerve; as a result, it may require another surgery to relieve the pressure on the nerve. Besides, due to insufficient exposure, its anastomotic effect is not as good as that of extensive incision surgery, and the risk of tendon re-rupture is higher. So, restoring the normal length, elasticity, and strength of the Achilles tendon and avoiding postoperative complications remain challenges for surgeons. Currently, there is no optimal treatment plan (4).

Bunnell's suture technique and Kessler's suture technique are commonly used. However, because most Achilles tendon ruptures caused by sports injuries are of the tearing type, with the broken ends being horsetail-like, which puts great restraints on these two methods (5). Krachow's suture technique is an interlocking whip-shaped suture method. During suturing, it has a continuous over—locking effect on both sides of the Achilles tendon, and after suturing, it has strong tensile strength and anti-splitting effect. Therefore, compared with other methods, it has a strong pulling force and a reliable suture. After suture, the number of suture threads at the broken ends is fewer, and the rejection is less. However, the influence of an interlocking suture on blood supply is a problem that can't be ignored (6).

The current study developed a new, simple overlapping suture technique that stacks the ruptured Achilles tendon, based on Bunnell's suture technique. This technique combines the advantages of both minimally invasive percutaneous surgery and extensive incisional surgery, thereby restoring the ruptured Achilles tendon and reducing the incidence of postoperative complications. Compared with Krachow suture, the blood supply of the Achilles tendon is effectively ensured. It's hypothesised that this surgical technique can achieve satisfactory clinical results.

## 2 Materials & methods

Inclusion criteria: (1) within 2 weeks after the acute Achilles tendon rupture; (2) 2–6 cm from the Achilles tendon rupture plane to the insertion point of the calcaneal tuberosity, measured by preoperative MRI or ultrasound; (3) under 60 years old; (4) no history of chronic Achilles tendinitis.

Exclusion criteria: (1) the site of Achilles tendon rupture is close to the Achilles tendon insertion or musculotendinous junction, or is combined with avulsion fracture of the insertion point; (2) the history of local hormone injection treatment for the Achilles tendon; (3) complicated with Achilles tendinitis or Achilles tendon calcification; (4) the patients can't cooperate with rehabilitation after surgery; (5) open Achilles tendon rupture; (6) having diabetes or immune-related diseases.

SPSS 19.0 is used to conduct the analysis. The data are expressed as mean ± standard deviation. A *p* < 0.05 is considered statistically significant. From May 2020 to May 2024, the longest follow-up was 4 years, the shortest was 1 year, and the average was 2 years. Of the 63 patients, 57 had systematic follow-up.

Arner-Lindholm scores include a comprehensive assessment of the patient's ankle joint range of motion, pain level, gait, and function at the last outpatient follow-up. According to the assessment, it's divided into four grades: excellent, good, fair, and poor. Excellent: the function of the ankle joint has fully recovered with no pain and normal gait, thus having the ability to perform various activities. Good: most functions have recovered, with little pain and basically normal gait, and most daily activities can be conducted. Fair: the function has been improved to some extent, with relatively obvious pain and unstable gait, and their daily activities are limited. Poor: the function has not recovered, with severe pain and obviously abnormal gait, and they are unable to carry out daily activities. At the last outpatient follow-up, all patients were functionally assessed according to Arner-Lindholm efficacy criteria.

The scoring criteria of the AOFAS foot-ankle scale consist of three dimensions: pain, function, and appearance. Specifically, it includes the following detailed scoring items: 1. Pain (40 points): it includes the evaluations of factors such as the aggravation and relief of pain, the duration, and the degree of pain. 2. Function (50 points): It includes the assessments of factors such as gait, range of motion, muscle strength, and activity limitations. 3. Appearance (10 points): It includes the evaluations of factors such as swelling, scars, deformities, and skin conditions (the higher the score, the better the function). Excellent: 90–100 points; good: 75–89 points; fair: 50–74 points; and poor: below 50 points. In the last follow-up, the AOFAS foot-ankle scale was used to score patients before and after the operation, and a paired-sample *T*-test was used for analysis.

The experimental protocol of the present study complied with the principles outlined in the Declaration of Helsinki and was approved by the Ethics Committee of XXX. All subjects provided a signed informed consent (2024KL-065)/(Date:2020.05.1).

### 2.1 Surgical technique

The surgery is usually performed under spinal anaesthesia. The patients lie prone. A pneumatic tourniquet is applied to the mid-thigh at a fixed pressure of 250 mmHg to achieve local ischemia in the surgical area.

#### 2.1.1 Preoperative detail preparation

Before surgery, touch the broken end of the Achilles tendon, and use a marker pen to mark it. The distance between each horizontal line is about 0.5 cm. After Achilles tendon restoration, these marks serve as guides for skin suture to ensure neat wound closure, which is important for preventing poor wound healing after surgery.

#### 2.1.2 The handle of the horsetail-like tear

**Figure 1:**
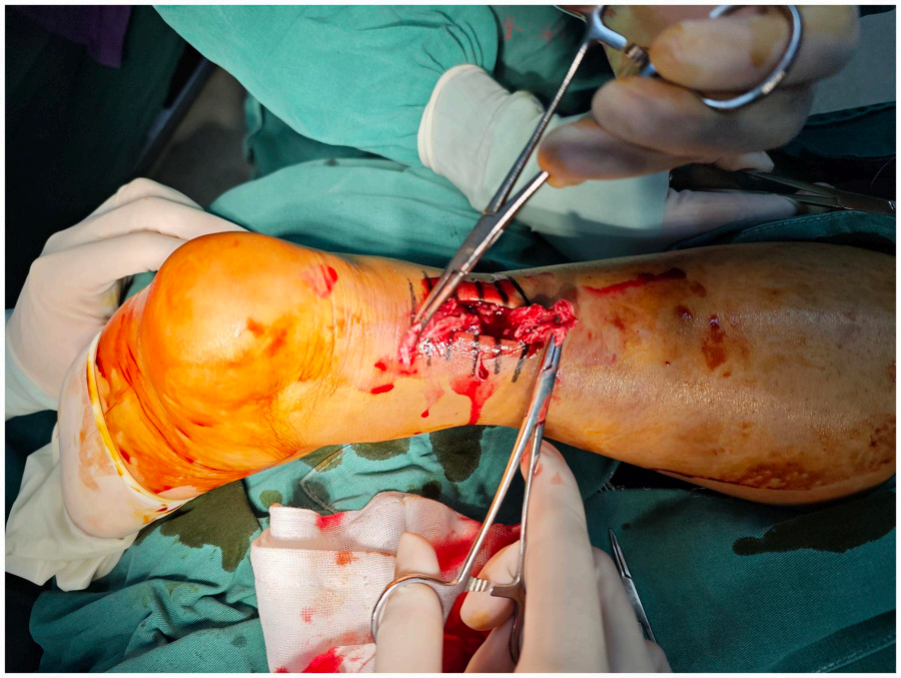
Sorting out the horse-tail-like rupture of the achilles tendon.

Make a 3-cm-long surgical incision slightly on the inner side to expose the broken end of the Achilles tendon. Then, locate and protect the sural nerve. The broken end of the Achilles tendon is represented as a horse-tail-like tear. Use a vessel forceps to arrange the Achilles tendons at the broken end and smooth them out. While smoothing, roughly overlap the two ends of the Achilles tendon according to the rupture condition (Figure 1).

### 2.2 Tighten the ends of the trimmed horse-tail-like achilles tendon

**Figure 2:**
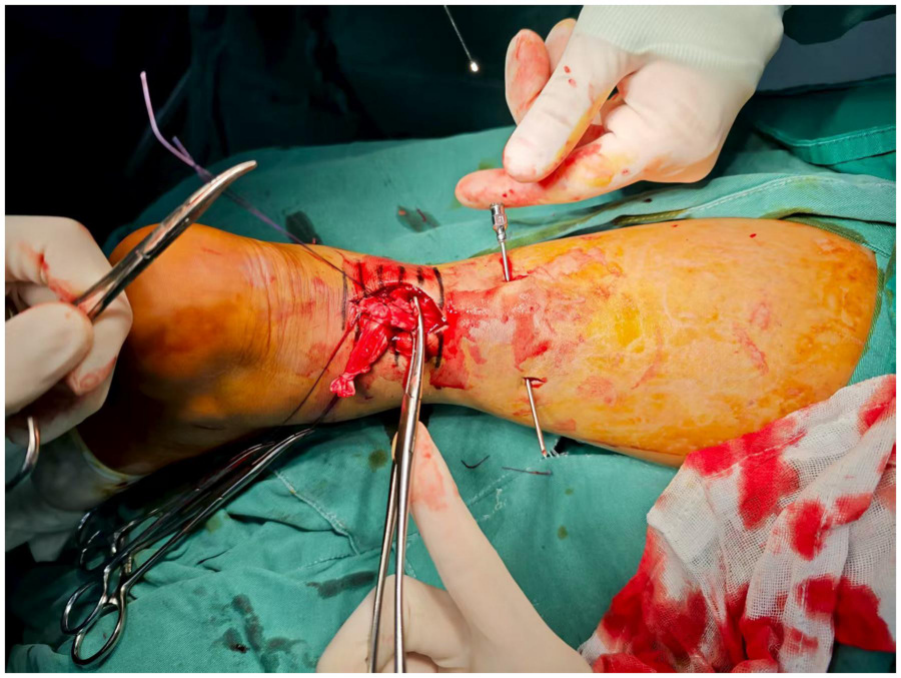
Pulling and looping the horse-tail-like end.

Use 2–0 absorbable suture to form suture loops on the trimmed tendon ends, with 2 or 3 sets of suture loops and traction on each side of the broken ends, respectively. Notably, while making loops and traction, the suture knots should be tied with moderate tension to avoid loosening. Otherwise, the traction suture may detach during subsequent overlapping of the broken ends, affecting the smoothness of the Achilles tendon suture (Figure 2).

### 2.3 Using the mainline suture of the achilles tendon/overlapping interweaving of achilles tendon ends

**Figure 3:**
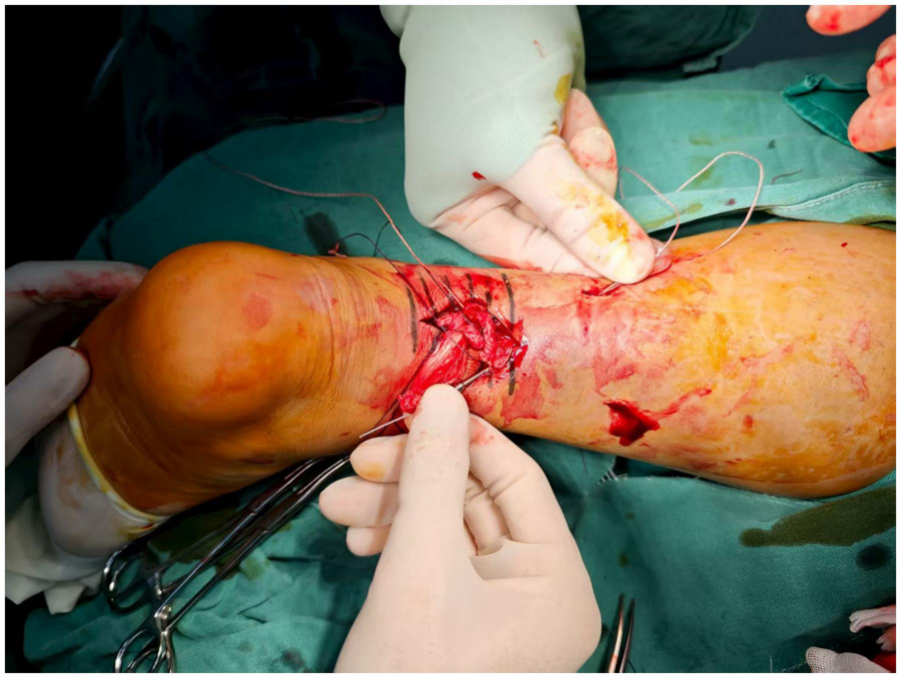
Using Bunnell's suture as the main suture.

**Figure 4:**
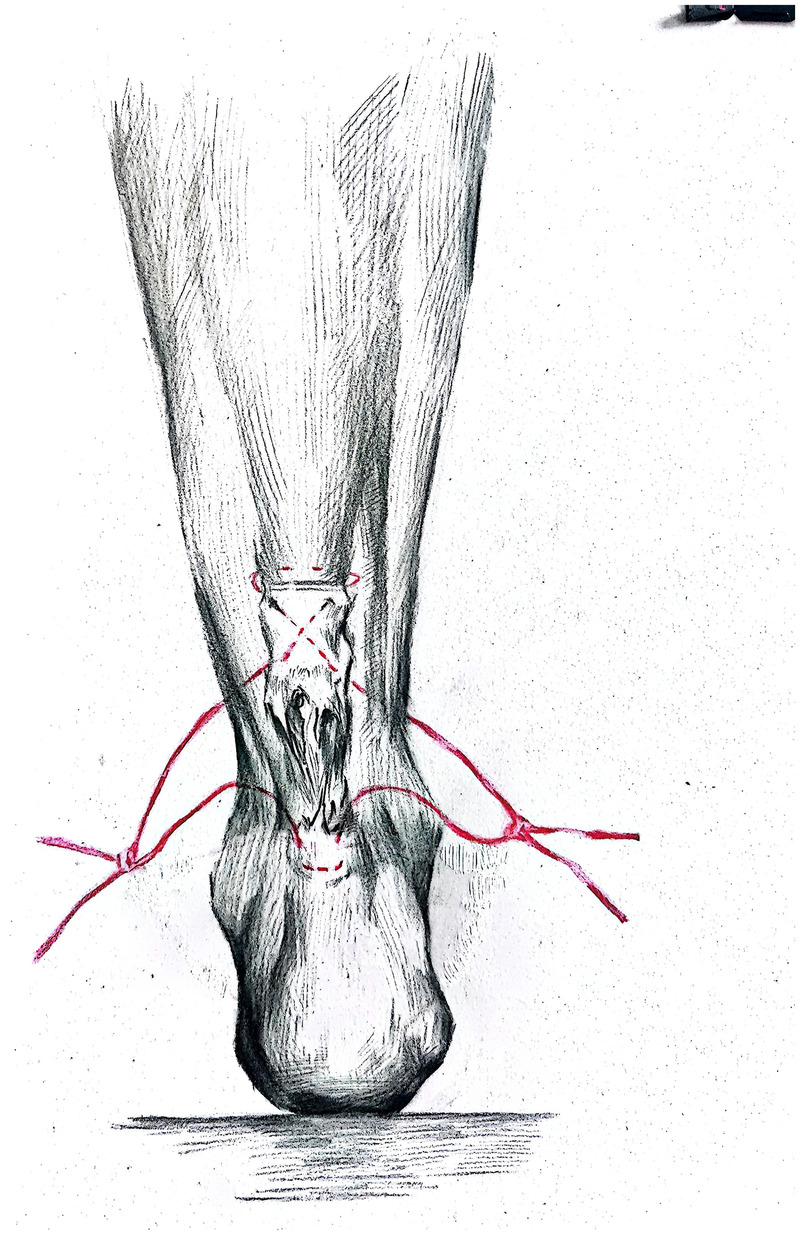
Schematic diagram of Bunnell's suture.

After the above steps are completed, use the modified Bunnell technique to suture the two ends of the Achilles tendon(5–0 suture). When puncturing and leading the suture at the broken end, the suture should be ensured within the paratenon (Figures 3, 4).

**Figure 5:**
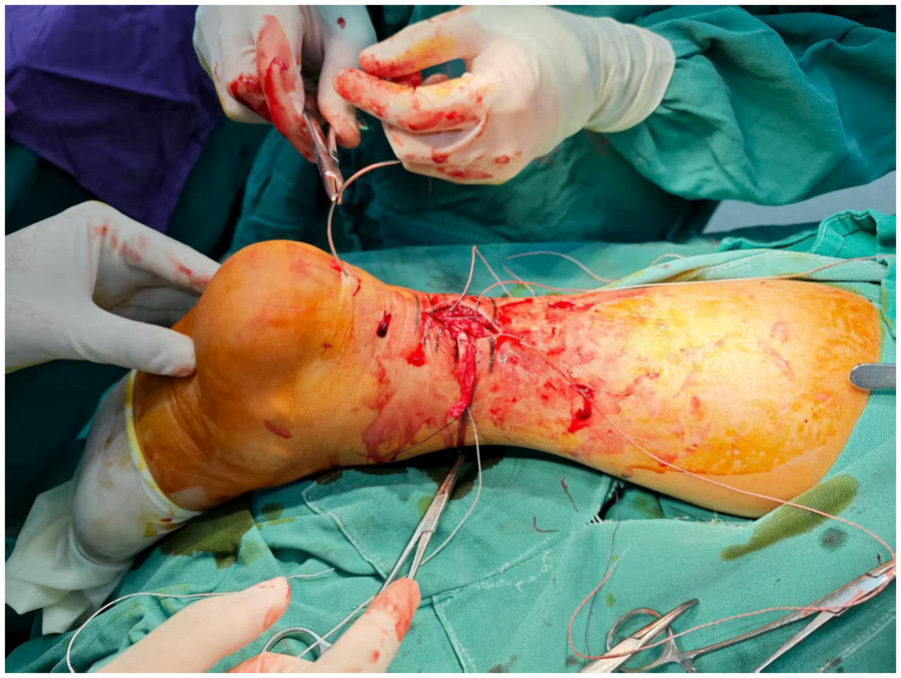
Using the large suture needle to overlap the broken ends of the horse-tail-like achilles tendon.

**Figure 6:**
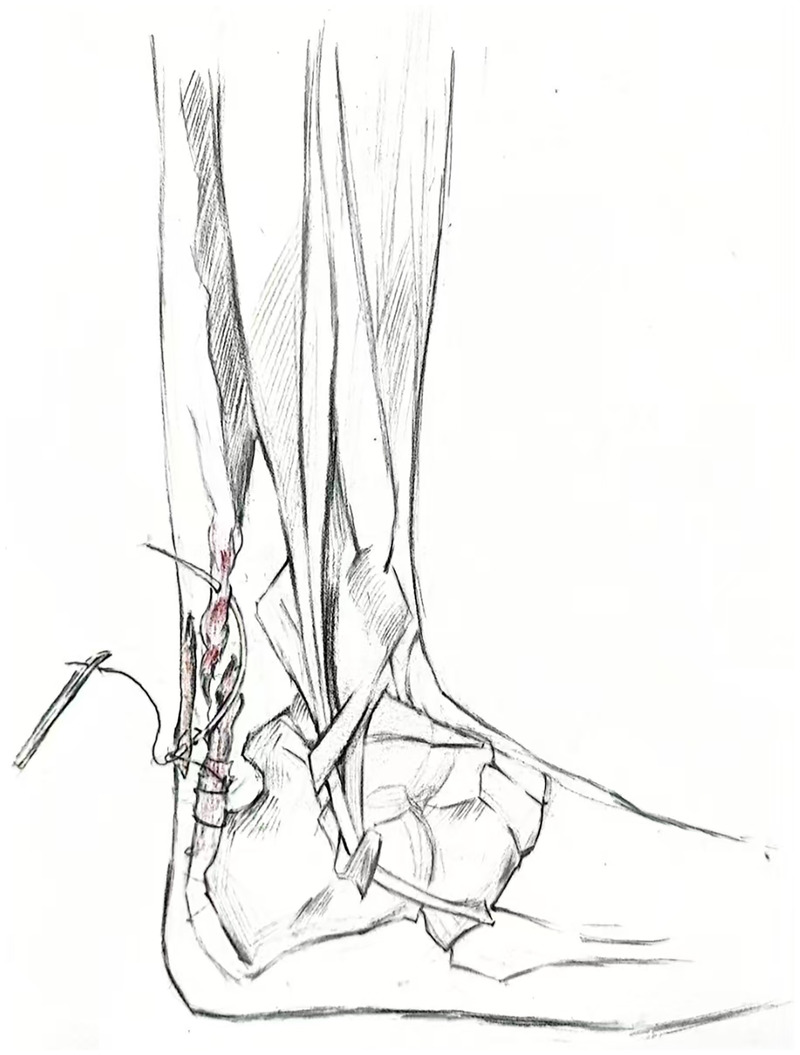
Schematic diagram of the overlapping horse-tail-like achilles tendon.

Before interweaving the Achilles tendon, the first step is to locate the upper and lower positions of the broken ends in the sagittal plane. Use a large suture needle to puncture and draw the “horse-tail” (looped with 2–0 suture) out of the skin from the base of the “horse-tail” at the lower end. Similarly, use a large curved needle to puncture and draw the “horse-tail” at the upper end out of the skin through the surface of the Achilles tendon and within the paratenon at its base. Temporarily leave these two sets of sutures on the skin surface for subsequent adjustment (Figures 5, 6).

It is important to note that the above procedure requires assessing the sural nerve's surface course to avoid iatrogenic injury. Several methods are commonly used for this purpose: 1. Identify the line connecting the midpoint of the lateral malleolus and Achilles tendon to the midpoint of the popliteal fossa as the approximate pathway of the sural nerve. Avoiding injury to this path can effectively prevent sural nerve injury (7); 2. Use a sharp scalpel to incise only the skin, and use small curved forceps to separate subcutaneous fascia from subcutaneous tissue to avoid nerve damage; 3. Select a medial incision at the site of the Achilles tendon rupture can effectively protect the sural nerve from damage; 4. When performing Bunnell's suture technique, all manipulations are conducted outside the sural nerve pathway.

### 2.4 Main knot

Tie the knot on the main suture and constantly adjust the tightness of the knot until the results of the Thomas test are negative. Therefore, at the same time, apply moderate tension to the 2–0 suture to ensure that the Achilles tendon is aligned smoothly.

### 2.5 Suture surrounding tissues

It is of utmost importance to suture the tissue around the tendon. The 3–0 absorbable sutures are often used for repair: wrapping the main suture around the main suture and the overlapping “horse-tail” tendon ends, then gradually suturing the skin according to the marks made before the operation to ensure the wound fits neatly.

### 2.6 Pull out the cord

**Figure 7:**
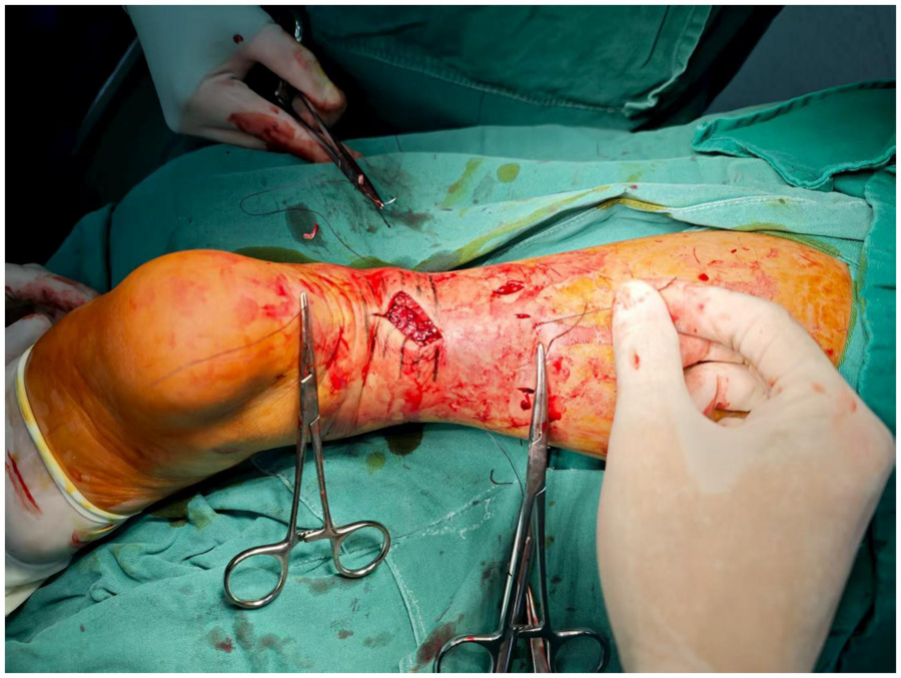
A horse-tail-like rupture of the lateral achilles tendon shown by MRI in the coronal plane and sagittal plane before the operation.

Finally, use a needle holder to remove the 2–0 sutures from both ends one by one, and re-smooth the folded Achilles tendon (Figure 7).

### 2.7 Postoperative rehabilitation

After the operation, gauze rolls are placed on both sides of the Achilles tendon to facilitate compression bandaging and prevent blood stasis. Within 4 weeks, a long-leg plaster splint should be used to immobilize the lower limb in a position with the knee and ankle fixed at 30°. During the immobilization period, patients should perform stretching and relaxation exercises for the lower limb muscles. After 4 weeks, it can be switched into a short-leg one. After 5 weeks, the plaster splint can be removed to perform flexion and extension training for the knee and ankle joints in bed. After 8 weeks, patients can walk with crutches in Achilles tendon boots, and the heel thickness can be gradually reduced. After 3 months, the patients can progress through training from slow walking to fast walking, jogging, and fast running. While practicing fast running, patients should perform heel-raising training, progressing from double-foot heel raising to single-foot heel raising. After 6 months, normal activities can be resumed.

## 3 Results

The 57 patients with acute Achilles tendon tears are all male, aged 24–50 years, with an average age of 39.8 ± 7.6 years. Causes of tears: 18 cases were due to strains while playing badminton, 11 cases while playing basketball, and 20 cases while playing soccer. There were 8 cases due to other causes. Among them, 7 cases had a history of Local injection therapy for the Achilles tendon using glucocorticoids or local anesthetic agents. (local injection of glucocorticoids or local anaesthetic agents). Before the operation, the MRI of their lower legs suggested a complete rupture within the range of 2–6 cm from the Achilles tendon insertion. The results of the Thompson Test were positive. The conditions of the soft tissue of the local skin at the broken end of the Achilles tendon were good. The surgeries were performed 2–10 days after injury, as shown in Table 1.

**Table 1:** The general information of patients.

Variables, Data	Patient
Age, y	39.8 ± 7.6
Injuries caused by playing badminton	18
Injuries caused by playing basketball	11
Injuries caused by playing soccer	20
The history of local closed treatment	7
Injuries due to other reasons	8

**Figure 8:**
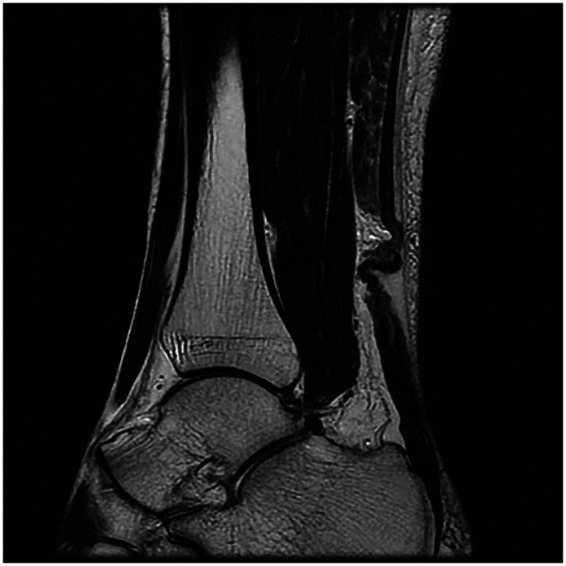
A horse-tail-like rupture of the lateral achilles tendon shown by MRI coronal plane and sagittal plane before the operation.

**Figure 9:**
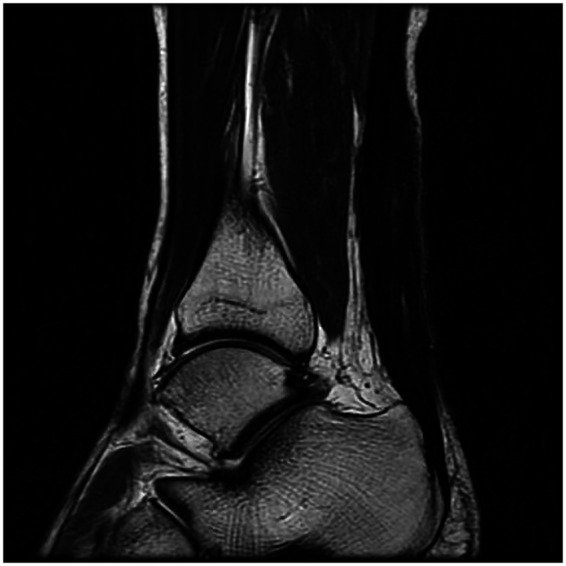
Indication of the continuity of the achilles tendon 3 months after the operation.

**Figure 10:**
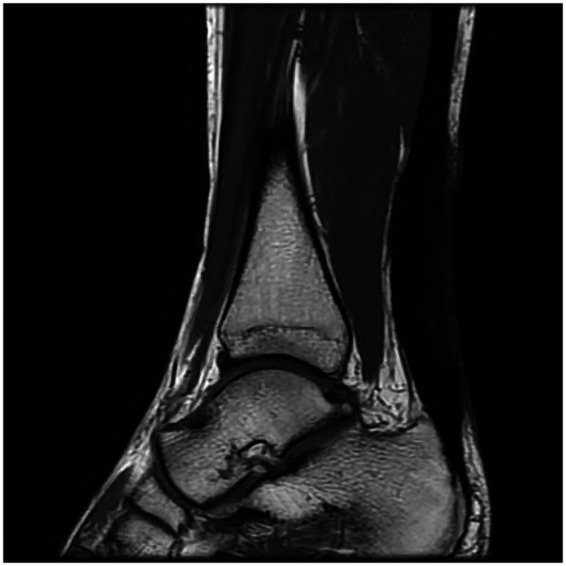
Indication of the continuity of the achilles tendon 6 months after the operation.

MRI coronal plane and sagittal plane of all patients showed a horse-tail-like rupture of the lateral Achilles tendon (Figure 8). The Achilles tendons of all patients were repaired by using a modified overlapping suture technique, and imaging follow-up at the third and sixth months after the operation indicated the continuity of the Achilles tendon (Figures 9, 10).

Among them, there were 49 cases with excellent results, 7 with good results, 1 with fair results, and 0 with poor results. Therefore, the excellent and good rate was 98.2%.

After surgery, the score is about 92.56 ± 6.71 points (95% CI, 90.81–94.30), which was significantly different from the score of 64.92 ± 9.08 points (95% CI，62.56–67.27) before the surgery (*p*-value is 0.00, which is less than 0.01). Among the patients, 52 had excellent scores after the operation, 3 had good scores, and 2 had fair scores. The excellent and good rate was 96.5%.

After the surgery, the muscle strength of the flexor plantar muscles of the affected ankle joint was (4.62 ± 1.7) grades, (95% CI, 4.17–5.06), compared with the healthy side [(4.80 ± 1.3) grades, (95% CI, 4.46–5.13)]. Therefore, when using the independent-samples *T*-test, there was no statistically significant difference (*P*-value = 0.527, greater than 0.05). However, there was a significant difference when comparing the muscle strength of the plantar flexor muscles of the affected ankle joint after surgery with that before surgery (3.2 ± 0.7 grades, 95% CI, 3.01–3.8) using the paired-samples *T*-test (*p*-value is 0.00, less than 0.01).

In the last follow-up, the maximum plantar flexion angle of the affected ankle joint is (37.1 ± 3.1)°, (95% CI, 36.2–37.9)°, compared with that of the healthy side, which is (38.3 ± 3.9)°, (95% CI, 37.2–39.2)°. The comparison using an independent-samples *T*-test was not statistically significant (*P*-value = 0.072, greater than 0.05). After surgery, the dorsiflexion angle of the patients' ankle joints is (18.3 ± 2.2)°, (95% CI, 17.7–18.8)°, and the dorsiflexion angle of the healthy sides is (18.2 ± 1.4)°, (95% CI, 17.8–18.5)°. There is no statistically significant difference when comparing using an independent-samples *T*-test (*P*-value = 0.773, greater than 0.05).

One patient suffered from delayed healing of the postoperative incision. After 2 weeks of enhanced dressing changes and local physical treatments, the incision healed. 3 weeks after the surgery, 2 patients suffered from re-rupture of the Achilles tendon due to accidental falls. Both cases received conservative treatment and, at the same time, enhanced postoperative daily care, with external fixation in the plantarflexion position for 8 weeks. One patient had a rupture of the opposite Achilles tendon after 2–4 years. There are no patients suffering from the symptoms of sural nerve damage. Among the patients who received follow-up, no complications such as infection, deep liquefactive necrosis of the Achilles tendon, skin necrosis, ankle joint stiffness, or Achilles tendinitis were found. During long-term follow-up, no postoperative Achilles tendon lengthening was found. The injury conditions and postoperative complications of the follow-up patients are shown in Table 2.

**Table 2:** Statistics of postoperative complications.

Postoperative complications	Patient
Postoperative damage to the sural nerve	0
Re-rupture	2
Rupture of the opposite side	1
Delayed healing of the incision	1

## 4 Discussion

Currently, there are arguments concerning the therapy of acute Achilles tendon rupture. Some literature suggests that there is no significant difference between conservative treatment and surgery (8). However, when the Achilles tendon ruptures, due to retraction of its distal and proximal ends, there is often a large defect in the Achilles tendon. During conservative treatment, the defect area is usually filled with fragile scar tissue, which increases uncertainty about the recovery effect and the risk of Achilles tendon rupture (3). Performing surgical treatment for young patients and those with athletic needs is currently the mainstream view. For a horse-tail-like Achilles tendon tear, the complete external shape of the tendon is lost after the tear, with the tendon bundles at the ends being interwoven, varying in thickness and length. This is an issue that cannot be ignored during surgery. The ultimate goal of treatment is the isometric recovery of the Achilles tendon and the minimization of postoperative complications.

Krackow suture without knot tension-relieving fixation for the treatment of the Achilles tendon. Among Achilles tendon suture methods, the Krackow suture method is widely used and relatively firm (9). However, it needs multiple overlock sutures on the inner and outer sides of the Achilles tendon. It can't be operated through percutaneous needle insertion, nor is it convenient to conduct a minimally invasive operation through a small incision at the broken ends. It usually requires open repair, and multiple overlock sutures can affect the blood supply of the Achilles tendon (10).

Compared with Krackow's suture technique, during the overlapping suture, there are only 5 small incisions (one 3-cm median incision, and the other 4 1-cm incisions). While using the Bunnell suture technique, the “ruptured horse-tail” is overlapped. Relevant literature indicates that under the same biological material conditions, the tensile strength of Krachow sutures is equal to that of Bunnell sutures. The course and surface projection of the sural nerve is predicted before the surgery. During suturing, the subcutaneous fascia was bluntly separated, which can effectively prevent damage to the sural nerve and is simple to perform. In addition, with reliable mechanical strength and minimal incisions, this technique not only reduces the risk of postoperative re-rupture but also lowers the incidence of surgical site infection. This improved technique compensates for the limitations of both large incisions and minimally invasive surgery, providing sufficient strength and preventing re-rupture. The research results show that the number of patients with sural nerve damage after operations was 0, and only 1 patient had postoperative delayed healing.

The research shows that there are 2 methods of Achilles tendon healing, that is, endogenous healing and exogenous healing (10, 11). The endogenous healing method involves the tendon tissues at the broken ends being in direct contact, and internal tendon cells proliferate and secrete collagen fibers, thereby promoting tendon healing and providing good biomechanics (4, 12). Exogenous healing methods depend on the ingrowth of peritendinous synovial cells and granulation tissue (13). And scar tissue adhesions form at the broken ends. Therefore, the biomechanical property reduces, and it's more likely to re-rupture (14). The use of an overlapping suture technique can ensure sufficient contact between the broken ends of the Achilles tendon, promoting endogenous healing. This method can flatten the “horse-tail-like” tear and repair the “tendon sheath,” wrapping the knot within the “tendon sheath.” This effectively avoids irritation to the posterior incision, thereby preventing complications from poor wound healing. The Bunnell sutures are widely used to provide sufficient tensile strength to prevent re-rupture (15). Among 57 follow-up patients in this study, only 2 patients had a re-rupture due to an accidental fall 3 weeks after the surgery, and 1 patient had a rupture of the opposite side. Current statistics indicate that this method is highly significant for treating patients with Achilles tendon rupture in clinical practice.

In theory, supported by current literature, this improved surgical technique uses minimally invasive methods to reduce the incidence of infectious complications; the Bunnell suture technique provides sufficient tensile strength, reducing the rate of re-rupture. The main result of this study is to propose a modified overlapping suture technique based on Bunnell's suture technique to treat horse-tail-like rupture and to verify its effectiveness by comparing AOFAS scores before and after surgery. The patient's plantar flexor muscle strength scores after the operation are better than before. Compared with that of the healthy side, there is no significant difference. Besides, after surgery, there are no differences in the dorsiflexion angle and maximum plantar flexion angle of the ankle joint between the affected side and the healthy side. These findings support the clinical application of this technique.

We acknowledge that this paper is a technical introduction with limited clinical statistics and sample size. Therefore, further observation of the long-term effect of this surgical technique is required. And there is no comparison between this study and other surgical methods. However, we would like to inform you that our preliminary results are inspiring. And we plan to analyze the long-term results of this technique and compare them with those of the mainstream repair technique, the Krackow suture. This will be the subject of future research projects.

## 5 Conclusions

In conclusion, the modified overlapping suture technique for treating horse-tail-like rupture of the Achilles tendon is convenient to perform, causes minimal trauma, and allows for rapid recovery. Moreover, it's a safe and reliable repair method to cure acute Achilles tendon rupture. However, this is a single-center retrospective study with a small sample size and a short follow-up period. There is no comparative study with other surgical methods. As a result, the above conclusions still need further study and improvement.

## Data Availability

The original contributions presented in the study are included in the article/Supplementary Material, further inquiries can be directed to the corresponding author.
